# Resilience at Work among Healthcare Professionals in Oncology during and beyond the Pandemic: Report from A Deliberative Multi-Stakeholder Reflexive Symposium

**DOI:** 10.3390/curroncol30070506

**Published:** 2023-07-21

**Authors:** Dominique Tremblay, Sophie Beaupère, Julien Biaudet, Patrick Castel, Béatrice Fervers, Christelle Galvez, Pascale Sontag, Susan Usher, Catherine Wilhelmy

**Affiliations:** 1 Faculty of Medicine and Health Sciences, Université de Sherbrooke, Longueuil, QC J4K 0A8, Canada; 2 Centre de Recherche Charles-Le Moyne, Longueuil, QC J4K 0A8, Canada; 3 Unicancer, 75013 Paris, France; 4 Cancéropôle Lyon Auvergne Rhône-Alpes, 69007 Lyon, France; 5 Sciences Po, Centre de Sociologie des Organisations (CSO), CNRS, 75007 Paris, France; 6 Département Prévention Cancer Environnement, Centre Léon Bérard, 69008 Lyon, France; 7 Centre Léon Bérard, 69008 Lyon, France; 8 Commissaire à la Santé et au Bien-Être, Quebec, QC G1S 2L2, Canada; 9 Centre de Recherche du Centre Hospitalier Universitaire de Sherbrooke, Sherbrooke, QC J1H 5N4, Canada

**Keywords:** resilience at work, oncology, deliberation, multi-stakeholder, well-being, healthcare professional

## Abstract

The chronic distress faced by healthcare professionals (HCPs) in oncology was exacerbated by the COVID-19 pandemic, heightening the need to improve their resilience. The *Entretiens Jacques Cartier* symposium provided an opportunity for participants from France and Quebec to share perspectives on resilience at work and discuss interventions at individual and organizational levels to support HCP health and well-being. Fifty-eight stakeholders were invited to the symposium, including HCPs, government decision-makers, researchers, and patient representatives. The symposium began with presentations on the nature of professional resilience at work in oncology and promising interventions developed in France and Quebec. Participants were then engaged in deliberation on how evidence and experiential knowledge could contribute to workplace strategies to strengthen resilience. Small-group reflexive sessions using the photovoice method, and an intersectoral roundtable, elicited the expression and deliberation of multiple perspectives on the nature and building blocks of resilience. Four main themes emerged from the discussions: (1) that resilience remains a muddy concept and can be associated pejoratively with “*happycracy*”; (2) that resilience must contend with bounded autonomy and captors; (3) that it relies on a sense of coherence at work; and (4) that patients play a role in improving HCP resilience. Stakeholders from healthcare systems in different countries view resilience at work as a means of equipping teams to handle chronic and punctual stresses in cancer care. The symposium emphasized the importance of better defining what resilience at work means and pursuing explorations of multicomponent interventions to support oncology HCPs and the patients they care for. The themes raised by participants at the symposium suggest pathways for furthering this exploration.

## 1. Introduction

The ability of healthcare professionals (HCPs) to manage and mitigate the adverse effects of facing difficult situations is essential to maintaining quality care for people living with and beyond cancer (PLWBC). Adversity arises from daily exposure to the suffering of patients, heavy workload and time pressures, workforce shortages, and administrative burdens [1]. The lengthy and profound disruption produced by the COVID-19 pandemic compounded a longstanding cancer crisis [2], aggravating scarcities of material and human resources and the siloed functioning of cancer care provision [3,4]. Consequently, oncology HCPs experience more stressful situations, are more dissatisfied with their work life, and more susceptible to physical and psychological problems.

Pre-pandemic studies found that oncologists report alarming rates of work-related stress syndromes, reaching a prevalence of up to 71% in Europe [5], and burnout symptoms that, in Canada, affect up to 73% of oncologists [6]. Literature reviews conclude that 32% of oncologists report emotional exhaustion, 24% report depersonalization, and 37% report low personal accomplishment [7]. Female oncologists seem to be at elevated risk of burnout [8]. A meta-analysis of burnout in oncology nurses reveals that 30% report emotional exhaustion, 15% report depersonalization, and 35% report low personal performance [9]. Psychosocial clinicians working in oncology settings (e.g., social workers, psychologists) show a similarly high risk of burnout [10]. Since 2020, there has been an exponential increase in studies on stress at work among oncology HCPs, responding to the exacerbation of work-related stress syndromes caused by the COVID-19 pandemic [1,3,11].

Evidence-based interventions are being implemented to generate HCP wellbeing [11,12,13]. For example, short cognitive behavioral interventions appear to be effective at increasing resilience and well-being in HCPs working at the frontlines during the COVID-19 pandemic [11]. However, in a scoping review of well-being interventions for healthcare workers, only 13 out of 8467 publications described interventions suitable for crisis situations [14]. The authors concluded that no high-quality theory-based interventions were available during the pandemic and that these could be useful in other crises. Multi-level interventions to move the burden of facing adversity from individual HCPs to organizational and national cancer plan levels have not yet appeared [1]. As healthcare and health system challenges multiply, resilience at work in oncology appears to be imperative and demands pragmatic solutions. The state of the science on interventions to better support HCP resilience capacity in oncology reveals the challenges of managing cross-cutting issues and mitigating adversity. It is a messy or so-called “wicked problem”, characterized by unclear problem formulation, a lack of immediate or ultimate solutions, and is potentially a symptom of other underlying problems [15].

### 1.1. Symposium Objectives

Given the severity and prevalence of these wicked problems internationally, we decided to organize a symposium at which multiple perspectives of resilience at work in oncology could be shared and pathways identified to build resilience capacity. The objectives driving the deliberative discussions were: (1) to disseminate knowledge about the nature of HCP resilience at work in oncology and promising interventions from the perspective of scientists, clinicians, patient associations, and government stakeholders; and (2) to create opportunities to use empirical research and examples of meaningful frontline initiatives to manage and mitigate adverse effects and learn for the future. Table 1 presents the symposium program.

### 1.2. Symposium Organization and Participants

In November 2022, the Université de Sherbrooke (Longueuil, QC, Canada) and the Centre Léon Bérard (Lyon, France) co-hosted a one-day symposium in the context of the 34th *Entretiens Jacques Cartier*, along with the Centre de recherche Charles-Le Moyne and the Cancéropôle Lyon Auvergne Rhône-Alpes. The deliberative symposium was planned and carried out to include comprehensive inputs from different health systems and disciplinary perspectives [16], using a network approach that is helpful when considering wicked problems [15]. Fifty-eight stakeholders were invited, including HCPs, government decision-makers, researchers, and representatives of PLWBC. They were selected based on their lived experience of oncology care or having a leadership position in cancer service organizations. Their participation offered a unique opportunity to share a plural understanding of problems and prompt a new set of pragmatic solutions that might not have otherwise been considered [17].

The formal presentations (see program in Table 1) were followed by roundtable discussions among experts from care, organization, and system levels on strategies to promote resilience at work and support HCP resilience capacity. Participants were then engaged in reflexive small group sessions (six to nine heterogeneous participants) adapted from the SHOWED technique for photovoice [18]. The reflexive approach was structured to reach symposium objectives, with questions such as ‘what do you see in the photo’, ‘how does it relate to resilience at work in oncology’, ‘what do you see as promising interventions to foster the resilience capacity of oncology HCPs’? These questions helped frame the discussion while leaving space for participants to freely share their perspectives and learn from each other. A PLWBC then presented on the patient’s role in HCP resilience at work in oncology in an interview-style plenary session. A video recording of vox pops with oncology HCPs [19] was also presented and a poster session promoted exchange and networking during the day. Three research professionals provided detailed notes from the presentations and from plenary and group discussions. This material was organized and condensed according to the symposium’s objectives [20] and was discussed among the authors to identify the key themes that emerged and their relevance for resilience at work. The symposium was conducted in French. Participants’ contributions cited in this report were translated by a co-author who participated in the symposium and has extensive experience in translation in the health field. The translations were validated by the relevant speakers.

## 2. Key Themes to Emerge from the Symposium

Although many ideas and solutions were generated throughout deliberations, we focused here on those that elicited most discussion during the symposium. The following four themes were considered consequential for efforts to generate resilience at work in oncology: (1) the muddiness of the concept of resilience and perceptions of resilience as a *happycracy*; (2) the impact of bounded autonomy and captors in oncology; (3) work-related sense of coherence as a contributor to resilience; and (4) the patient’s role in team resilience in oncology. 

### 2.1. Theme 1: The Muddiness of the Concept of Resilience and the Happycracy

One of the primary objectives of the symposium was to clarify the nature of resilience at work in oncology. Participants were familiar with the multiple definitions of the concept of resilience in the scientific literature of various disciplines, including health care, where it refers in general terms to the capacity to face difficult situations and maintain the quality and safety of care. Beyond that common thread, symposium participants characterized the concept as “*muddy*” and felt it was used in different situations for different reasons. Resilience appears to be a positive value-laden concept, and HCPs who find it difficult to achieve can be made to feel inadequate. The word “*happycracy*” was used to describe the expectations associated with resilience. Resilience can be a context-dependent process or an outcome. Participants emphasized that it cannot be taken for granted and varies over time. On a daily basis, clinicians work with people affected by the burden of a potentially fatal disease, the suffering of PLWBC and their loved ones, and the end-of-life trajectory. One symposium participant in a managerial role stated that “*We all know what we are getting into when we choose to work in oncology. But the expectations can become overwhelming. We need to adapt to the explosion in number and sophistication of cancer treatments and the growing demands on cancer services in the context of limited resources and administrative constraints*”. A number of participants expressed that these workload and time pressures made it all the more important to use rare moments of downtime to recover one’s calm, talk to colleagues, and remember why one chose to work with people affected by cancer. These multiple strategies were seen to prevent the loss of meaning at work during difficult times.

The pandemic made it even more difficult for HCPs and managers to ensure reasonable delays from diagnosis to treatment, provide a prompt response to whole patient needs, and prevent disruptions to the continuity of care. Participants related to the statement by a clinician participant at the symposium that: “*We cannot let adversity reduce our longstanding efforts to nothing. However, a study in France shows that near half HCPs view their work as a burden, not a source of personal fulfillment*”. Resilience calls for a participatory approach, where managers trust their oncology team and where this approach serves to maintain or restore individual and collective self-esteem. An important take home message emerged from the discussion that resilience should not fall into the tyranny of “*everything is OK*”, the so-called *happycracy* that makes joy at work an obligation. HCPs should have the space to express the challenges they face and benefit from the support of their team and organization. Seeking support when needed helps to increase the capacity for resilience.

### 2.2. Theme 2: HCPs, Bounded Autonomy and Captors

One clear challenge to emerge in symposium discussions was related to the superspecialization of cancer care that makes it difficult for individual providers to find the space to define their own resilience. Participants considered that oncology HCPs need to develop a greater sense of diversity and inclusion to overcome the bounded autonomy related to the dynamics of a rigid scope-of-practice. No one can singlehandedly manage the complexity of cancer care, but some still come to act as “captors” along the cancer trajectory, building invisible walls around the patient. Collaboration within and between teams has yet to fully develop to support shared leadership that brings together senior officials, professionals, lay care providers, and patients. All stakeholders have some degree of autonomy, but tension appears at the boundaries. Professional autonomy is based on scientific, disciplinary, and clinical judgment. A cancer specialist stated that: “*The hegemonic status of specialized care encourages oncology teams to follow patients even long into survivorship, and the transition to front-line teams is often difficult, as these teams do not feel well trained and equipped for survivorship follow-up. Patients can thus experience a sense of abandonment when they experience incomplete transitions*”. Discussion at the symposium related the fragmentation of the cancer pathway to this bounded autonomy, which creates power struggles between captors (specialized teams) and other professionals, and may adversely affect patient partnership and the integration of non-profit community organizations into the cancer pathway. The result is a captive clientele, which is sub-optimal for patients and leaves oncology teams overwhelmed and the healthcare system vulnerable. 

### 2.3. Theme 3: Work-Related Sense of Coherence

A practical suggestion that emerged from discussions, while not easy given the multiple providers and levels involved in cancer care, is to support interventions to prevent a loss of sense-making. A front-line manager emphasized: “*Actions need to align with discourse. Once managers and policy makers have promised something, it is important to HCPs and teams that they keep their promises*”. A patient representative agreed and insisted that the same applies to interactions between HCPs and PLWBC. The difficulties experienced come mainly from three sources: the difficulties of announcing a cancer diagnosis or progression, the complexity of treatments, and misunderstanding or poor communication of objectives among team members. Difficulties arise when there is too much uncertainty about decisions and when loss of meaning impedes decision-making. Helpful strategies described during the symposium include raising awareness and training managers on the importance of participatory approaches and collective work. Teamwork within and between teams, supported by a visible and committed manager, protects time and collaborative spaces and enables the expression of a plurality of points of view in a non-hierarchical way and the search for a common perception of a problem and consensus on potential solutions.

### 2.4. Theme 4: The Patient’s Role in Improving Cancer Team Resilience

One of the very interesting contributions of the symposium was to provide practical examples of how patients can contribute to improving HCP resilience capacity. A PLWBC representative with extensive lived experience stated that “*Patient engagement can contribute to HCP resilience in oncology…There are many lost opportunities regarding the potential contribution of patient and public engagement*”. While not all patients may be willing or able to adopt the patient-partner role, some find that this type of participation in the battle against cancer makes sense and brings them knowledge and understanding they would not have otherwise. A number of symposium participants mentioned the importance of patient partnership for team resilience. The patient is the only one to experience transitions across the entire cancer trajectory: from home to hospital and back to ‘new normal’ life, with cancer always in the background. Patients contribute to the coherence of HCP work: “*Patients are able to restore meaning. The bounded autonomy previously mentioned is not only a professional matter. To properly define the scope of partnership, patients must understand and situate their contribution in the overall picture and clarify what our influence is or could be*”. From this perspective, acknowledging the patient’s role is a step toward greater resilience in cancer teams: when there is a loss of meaning within teams, patients can bring this sense back by identifying what is most important. Deliberations during the symposium raised the idea that the burden of health has been shifted too far onto clinicians, but that PLWBC and the public also have a role. As one participant put it: “*When responsibility is better shared, there is room for a more equal relationship between clinicians and patients. Getting to better know ourselves and each other raises trust and increases each of our ability to support health and well-being*”. The importance of considering the whole person does not apply only to patients; it also entails considering HCPs as more than medical science experts. 

## 3. Next Steps

The themes generated through discussion at the symposium appear to be highly relevant to better supporting capacities for resilience at work in oncology. Each suggests a number of strategies and research directions in line with evolving literature.

### 3.1. Resilience as an Individual or Group Responsibility and Burden

Participants reflected on the growing efforts to define resilience [21]. First developed in physics to describe a metal’s resistance to pressure, it is now firmly embedded in public and policy discourse. Interestingly, resistance to pressure did not figure prominently in the various definitions that emerged during symposium deliberations. Resilience has been described as a conceptual nebula juggling with multiple disciplinary boundaries [22]. It became a buzzword during the multiple waves of the pandemic. Long seen as a positive personal characteristic, resilience is also considered an attribute of teams, organizations, and system processes [23,24]. Without neglecting individual HCP responsibility for self-care, some of the burden for resilience capacity can be removed from their shoulders if resilience is understood from an ecological standpoint [1,25]. For some, resilience refers to joy at work, where sense of humor is emphasized to minimize the adverse effects of difficult situations [26]. Resilience can be addressed as a boundary concept [27], which provides opportunities for heterogeneous individuals to negotiate how to define and collectively manage adverse clinical or administrative situations and learn for the future; in so doing, they make explicit and share their representations of the proper functioning of their department or institution. For others, the positive ideology associated with resilience imposes a “tyranny of happiness”, where resilience is prescribed, becomes the norm [28], places responsibility for managing situations on the individual and thus generates stigma among HCPs who have difficulties with the challenging oncology context. A clearer definition of resilience at work might align with requests from HCPs to: “… hear me, protect me, prepare me, support me and care for me” [29] (p. 2134). Interventions targeting these requests can contribute to overcoming the well-recognized reluctance of many HCPs to ask for help.

### 3.2. Recognize the Multiple Notions of “Good Care”

Issues around bounded autonomy and captors warrant further exploration in order to assure quality and safety and more integrated person-centered care with smooth transitions along the cancer trajectory. Autonomy is bounded by professional obligations, the singularity of each PLWBC’s health and life objectives, and accountability systems. All share a concern for “good care” but define it in different, and not always complementary, ways. In professional practice, autonomy based on scientific, disciplinary, and clinical judgment is the main basis for decision-making, while a patient’s autonomy comes from their lived experience of health and cancer. Both can be affected by organizational controls and structures that influence the conditions under which decisions are made [30]. The boundaries of autonomy can help to define the scope of action one feels confident and competent to take. However, for specialized oncology HCPs, effective autonomy can involve becoming captors of the care trajectory [31], building invisible walls around the patient and compromising integrated care [32] and the contribution of other providers. The different conceptions of what is “good care” and variable HCP commitment in the patient’s trajectory, depending on acceptance of, and potentially competition for, the captor role, can create difficulties for coordination [31], with consequences for the individual and collective experience of HCPs and their resilience. There is a need to better understand and develop strategies to manage tensions between bounded autonomy and interdependence in order to improve capacity to manage adverse situations, minimize their impact, and learn for the future.

### 3.3. The Relational Aspects of Meaning and Motivation

The concept of sense of coherence appears in Antonovsky’s salutogenic model [33]. Work-related sense of coherence (Work-SoC) refers to a global ability to view one’s internal and external environments as comprehensible, manageable, and meaningful [34]. These perceived dimensions depend on professional characteristics (personality, behaviors, experience) and oncology practice environment (job demands, resources, structures, processes). Comprehensibility relates to the extent to which a professional perceives the work situation to be structured, consistent, and clear. This emphasizes the importance of proximity between managers and their teams, especially in times of crisis. It is critical that leaders understand the sources of concern, assure HCPs that their preoccupations are recognized, and work to develop approaches that limit as much as possible the adverse effects of these preoccupations [29]. Manageability in oncology is improved by interdisciplinary teamwork, which provides resources to cope with demands and improves cancer care responsiveness from the patient’s perspective [35]. Meaningfulness helps maintain the motivation and engagement of HCPs in oncology. Maintaining meaningfulness is a priority given that a recent survey of 1520 HCPs from 101 countries reveals a 66% increase in the proportion who report not being able to perform their job compared with the pre-COVID-19 period [36]. The pandemic has forced social distancing and the rapid adoption of telemedicine, both of which risk jeopardizing the relationship between professionals and patients [37]. Rebuilding a sense of coherence at work may depend on the capacity to support relational and cognitive proximity within an integrated cancer network [38].

### 3.4. The Patient’s Role in Improving Cancer Team Resilience

Patient engagement has many impacts. Particularly when paired with HCPs, patient navigators have the potential to improve cancer system responsiveness [39]. Responsiveness can be viewed as an indicator of the quality of bidirectional interactions between healthcare users and HCPs [40,41] that is associated with prompt attention, person-centered response, quality of communication, and quality of the care environment. Patient participation supports the benefits of a better mutual understanding between practitioners and patients of one another’s reality [42]. The joint action of professional and experiential expertise may represent a mitigation strategy to responsiveness gaps, which in turn reduces adversity. In a report from leadership roundtables, one solution to move toward a more resilient healthcare system was patient-centricity as an approach “that will give the health care system, the resilience it needs” [3] (p. 1735). The symposium proposed a step forward, highlighting the patient’s role in contributing to HCP resilience. Additional research on the effects of patient and public engagement [43] on work adversity in oncology and HCP health and well-being is warranted.

## 4. Conclusions

The symposium generated insights on resilience at work for people in oncology and emphasized the importance of maintaining capacities in the face of adversity. This report focused on four key themes that emerged in deliberative discussions between stakeholders from France and Quebec who have first-hand knowledge of managing adversity in daily practice. Participants from France and Quebec described similar challenges, despite differences in their healthcare systems and the organization of cancer care—Quebec’s health system is publicly funded, while France has a public-private system [44]. 

Symposium participants shed light on supportive interventions to support resilience in the real-world practice of clinical oncology teams. They also pointed to interventions to promote physical health and well-being that include interventions to reduce stress such as reorganizing workflow and reducing administrative constraints, developing continuing education on resilience, and expanding conceptions of PLWBC’s contribution to HCP resilience. The symposium emphasized the importance of better defining what resilience at work means and pursuing explorations of supportive interventions. The multifactoral origin of work-related stress in oncology HCPs requires that resilience be conceptualized at a collective level and that a multidimensional response be co-designed using complementary approaches to reinforce capacities for resilience at work. 

## Figures and Tables

**Table 1:** Symposium program summary.

Sessions	Themes and Speakers
Welcome	34th *Entretiens Jacques Cartier*: a longstanding opportunity Dominique Tremblay, Université de Sherbrooke; Centre de recherche Charles-Le Moyne (Longueuil, QC, Canada)
	Mylène Honorat, Cancéropôle Lyon Auvergne Rhône-Alpes (Lyon, France)
Introduction	Why is resilience at work important in oncology? Cloé Rodrigue, Centre de recherche Charles-Le Moyne (Longueuil, QC, Canada)
	Sophie Beaupère, Unicancer (Lyon, France)
Formal presentations followed by plenary deliberative discussion	How resilience draws on institutional and professional logics Patrick Castel, Centre de sociologie des organisations, SciencesPo/Centre de Sociologie des organisations (Paris, France)
	Resilience in cancer care: example of virtual symptom monitoring and follow-up Thomas Joly-Mischlich, Department of Pharmacy, Centre intégré universitaire de santé et de services sociaux de l’Estrie—Centre hospitalier universitaire de Sherbrooke (Sherbrooke, QC, Canada)
	Catherine Wilhelmy, Patient-Partner, Centre de recherche du Centre hospitalier universitaire de Sherbrooke (Sherbrooke, QC, Canada)
	Situation of adversity? Strategic and tactical challenges to building resilience Dr Fadila Farsi, Centre Léon Bérard; Regional cancer network Auvergne Rhône-Alpes (Lyon, France)
	Team resilience at work in oncology: supporting each other to care better Dominique Tremblay, Université de Sherbrooke; Centre de recherche Charles-Le Moyne (Longueuil, QC, Canada)
Roundtable discussion followed by Q-R exchanges	Challenges and promising strategies at different levels of the healthcare system Facilitator: Dominique Tremblay, Université de Sherbrooke; Centre de recherche Charles-Le Moyne (Longueuil, QC, Canada)
	Susan Usher, Commissaire à la santé et au bien-être (Quebec, QC, Canada)
	Sophie Beaupère, Unicancer (Paris, France)
	Christelle Galvez, Centre Léon Bérard (Lyon, France)
	Élisa Gélinas-Phaneuf, Réseau de cancérologie de la Montérégie (Greenfield Park, QC, Canada)
	Pascale Sontag, Association française des infirmiers(ères) de thérapie cellulaire, hématologie, oncologie et radiothérapie; Centre Léon Bérard (Lyon, France)
Presentation followed by deliberative discussion	Beyond the romantic illusion: the dark side of resilience Julien Biaudet, Cancéropôle Lyon Auvergne Rhône-Alpes (Lyon, France)
Reflexive small group sessions	Intersecting perspectives on situations of adversity and actions to support resilience at work in oncology All symposium attendees
In-depth interview	Dialogue between a researcher and a person living with and beyond cancer Dominique Tremblay, Université de Sherbrooke; Centre de recherche Charles-Le Moyne (Longueuil, QC, Canada)
	Catherine Wilhelmy, Patient-Partner, Centre de recherche du Centre hospitalier universitaire de Sherbrooke (Sherbrooke, QC, Canada)
Conclusion	Concluding remarks and next steps Dominique Tremblay, Université de Sherbrooke; Centre de recherche Charles-Le Moyne (Longueuil, QC, Canada)

## Data Availability

The data presented in this paper are available in this article.
